# Health system adaptation to extreme weather events in Australia: A scoping review

**DOI:** 10.1016/j.joclim.2025.100443

**Published:** 2025-04-28

**Authors:** Rupert Legg, Jason Prior, Erica McIntyre, Edgar Liu, Mikaela Tracy, Leona Tan, Angela Dawson, John Richmond, Clare Perry

**Affiliations:** a Research Institute for Innovative Solutions for Well-being and Health (INSIGHT), Faculty of Health, University of Technology Sydney, Ultimo, NSW 2007, Australia; b Institute for Sustainable Futures, University of Technology Sydney, Ultimo, NSW 2007, Australia; c Institute of Earth System Sciences, Leibniz University Hannover, Hannover, Germany; d City Futures Research Centre, University of New South Wales, Sydney, NSW 2052, Australia; e Sheffield Centre for Health and Related Research (SCHARR), University of Sheffield, Sheffield S10 2TN, UK

**Keywords:** Health systems, Extreme weather events, Climate change, Adaptation, Resilience, Australia

## Abstract

**Introduction:**

The increasing prevalence and severity of extreme weather events is likely to present challenges for the systems in which humans operate. This review investigates how the health system in Australia, a region heavily affected by bushfires, floods, droughts and extreme heat, is adapting to the risks presented by extreme weather events and how these adaptations are being evaluated.

**Methods:**

By searching Medline, PsycINFO, Scopus and Web of Science for peer reviewed literature reporting on health system adaptations, 33 articles published between 2014 and 2023 were identified for inclusion.

**Results:**

Primarily, articles documented adaptations that, consistent with the World Health Organization's health system building blocks, focused on: the health workforce; health information systems; leadership and governance; and service delivery. Little attention was placed on access to essential medicines and health system financing. It was also most common for adaptations to address flooding, extreme heat, bushfires, and storms, reflecting the impact of such events in Australia. Adaptations tended to result in beneficial outcomes, including improved workforce capability, better health outcomes, reduced demand on and risk of overburdening the health system, lowered costs, and greater financial stability. However, how these elements come together to build health system resilience is unclear and barriers remain that reduce the effectiveness of adaptations.

**Conclusion:**

To ensure that Australia's health system is resilient to extreme weather events, future adaptations should focus particularly on access to essential medicines and financing, while future research should evaluate the outcomes of adaptations in a consolidated and systematic way.

## 1 Introduction

Climate change is causing global weather patterns to shift, temperatures to rise, rainfall patterns to alter, and the frequency and severity of extreme weather events (EWEs) to increase [1]. EWEs are “the occurrence of a value of a weather or climate variable above (or below) a threshold value near the upper (or lower) ends of the range of observed records of the variable” [2], including heatwaves, coldwaves, droughts, storms, flooding, bushfires, among others [3]. Such events present risks to society's systems and their functioning, disrupting human livelihoods, adversely affecting health, damaging infrastructure, and impeding essential services [4].

Health systems, consisting of multiple complex components like service delivery, health workforce, health information systems, access to essential medicines, leadership and governance, and financing [5], are particularly vulnerable to EWEs. EWEs have had documented adverse impacts on health systems, including damaging health infrastructure, disrupting the transportation or delivery of medical products, and reducing the capacity of the health workforce to function and services to be delivered [4,6,7]. Given the complexity and interdependency of health systems, impacts may be wide-ranging and cascading.

In recent years, Australia has experienced several EWEs, such as the extreme bushfires across the East Coast in 2019 that were immediately followed by several flooding events, droughts, and even more bushfires [8]. Such EWEs are predicted to become more frequent and severe [9,10], and will place considerable pressure on Australia's health systems even when occurring in isolation [10]. For instance, EWEs that result in increased numbers of ambulance call-outs and emergency department presentations create bottlenecks in the provisioning of medical services elsewhere [4,11]. Further, cascading crises, where one or more secondary events occur after the initial event and become interconnected, are also predicted to occur more frequently and will have impacts on health systems greater than their parts through the continued disruptions they cause [12].

Australian state and federal governments have developed strategies to mitigate the increasing threat posed by EWEs. The *National Strategy for Disaster Resilience 2011* sought to prepare the country for EWEs by moving away from response-based recovery approaches towards prevention, preparedness, and mitigation [13]. While the *Strategy* did not focus on health systems directly, the *National Health and Climate Strategy 2023* later sought to “build a climate-resilient health system and enhance its capacity to protect health and wellbeing from the impacts of climate change” [14]. The Australian Government's *National Climate Risk Assessment* and *National Adaptation Plan*, once finalised in 2025, will aim to build on these strategies and encourage collaboration across sectors [15]. How these strategies have resulted in actual changes in health systems in Australia remains to be documented.

Examining how health systems in Australia are adapting to EWEs can reveal how resilient they currently are. Health system resilience is “an emergent property that allows health systems to maintain core functions by withstanding and adapting to shocks while also leveraging shocks as opportunities for growth and improvement” [16]. Resilience can exist to three different degrees: absorptive, adaptive, and transformative [17]. Absorptive capacity is the potential for the health system to continue delivering care during an EWE, using the same level of resources and capacities. Adaptive resilience refers to a health system's ability to function at the same level as normal with fewer and/or different resources and capacities. Finally, a health system that has transformative resilience changes and grows during and after an EWE. However, each implemented adaptation strategy does not necessarily contribute to a health system becoming more resilient, so evaluating their outcomes is also a must.

Research on how health systems are adapting to EWEs is growing. Previous empirical efforts have documented case studies in Australia and England [18,19], while reviews have attempted to holistically record health system adaptations in the Asia-Pacific region [16] and globally [20,21]. There remain knowledge gaps surrounding how health system adaptations are being evaluated and assessed, a point that this study seeks to address through the following research questions:

1. In what ways has the Australian health system (at various scales, from hospitals and local health districts to the national level) been documented to be adapting to (preparing for, responding to and recovering from) EWEs in the literature?
2. How have these documented adaptations been evaluated in the literature?
3. Considering this evidence, to what extent are these adaptations contributing towards Australia's health system becoming more resilient to EWEs?

A scoping review was selected as the appropriate approach to answering these questions as the intention was primarily to identify and map the overall distribution and focus of research, rather than report on specific data or statistical outcomes, as per systematic review and meta-analyses.

## 2 Method

The scoping review method reported in this paper has been registered with the Open Science Framework [https://doi.org/10.17605/OSF.IO/R4C6W] and follows PRISMA-ScR guidelines [22].

### 2.1 Eligibility criteria

Peer-reviewed, original quantitative and qualitative research published in English from 1 January 2014 to 1 December 2023 (date of initial search) and reporting on empirical data relating to the Australian health system and its adaptation to extreme weather was included. After conducting a preliminary horizon scan of grey literature on Australian health system adaptations to extreme weather, we determined that much of the literature was documenting plans to be implemented, leaving a gap in knowledge regarding their success. As such, we decided not to include grey literature in this scoping review and to instead focus predominantly on how adaptations are being evaluated. Consequently, it is possible that the most recent and cutting-edge adaptations currently being implemented in Australian health systems are not captured in this present scoping review. The date was set to 2014 to capture only the most recent and developing adaptations being implemented within health systems. Review articles, conference papers, and dissertations were excluded, as were studies focusing solely on the impacts of extreme weather on the health system in Australia and not investigating how the system responds; for instance, see Jegasothy et al. [11]. Articles that either evaluated how prepared a particular component of the health system was for future EWEs or examined how a component responded to past EWEs while identifying what form responses or adaptations were taking within this were included.

### 2.2 Information sources and search strategy

Medline, PsycINFO, Scopus and Web of Science were searched, followed by a manual search for original research included in the reference lists of eligible articles. The search strategy was divided into four groups, each with terms oriented around extreme weather, health systems, adaptation and location. Details of the specific search terms are presented in Table A1 (see Appendix).

### 2.3 Screening and study selection

**Fig. 1:**
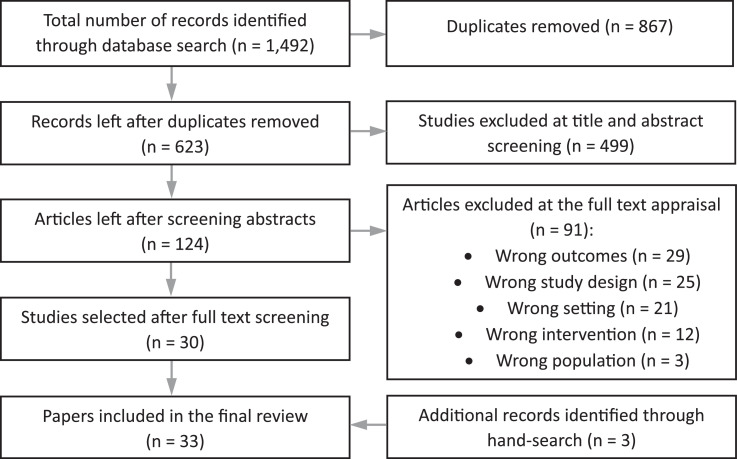
PRISMA diagram of the search and screening process.

Search results were loaded into EndNote and duplicates were removed. Using Covidence, RL and CP independently screened the titles and abstracts against the inclusion and exclusion criteria. Next, the full texts of remaining articles were screened independently by RL, MT and CP for inclusion (Fig. 1). The references of included articles were also examined through a manual search to determine if any articles were missed in the database search that could be included.

### 2.4 Data extraction

The following data were extracted from each study independently by three authors (RL, MT and CP), with discrepancies discussed between the authors to reach a consensus: title, authors, year published, location in Australia, design and methods, extreme weather type, health system component studied, scale of health system component, adaptation strategy implemented, reason provided for implementing the adaptation, and the outcome of the adaptation. The health system component addressed by the adaptation strategy was determined using the definitions and examples provided by the World Health Organization's (WHO) report on health system building blocks [5].

### 2.5 Quality assessment

The Mixed Methods Appraisal Tool (MMAT) was chosen for the quality assessment as it provides a means of assessing quantitative, qualitative, and mixed methods studies [23]. Each study was allocated to the relevant study design category for appraisal. The answer to each of the five questions pertaining to that study's design was categorized as “yes” (Y), “no” (N) or “cannot tell” (CT). Given that the calculation of an overall score is discouraged, a sensitivity analysis is visually depicted in Table A2.

### 2.6 Synthesis of results

Given the wide variety of methodological approaches and disparity of outcomes reported across the included studies, we opted to apply a meta-narrative synthesis on the extracted data [24]. Similarly, to Rameshshanker et al. [16], the data were thematically organised to create a conceptual model outlining the various ways health systems in Australia are adapting to extreme weather and how they have been evaluated. This process involved the collaboration of the entire review team.

## 3 Results

Thirty-three articles were included in the review and are summarised in Table 1 and provided more completely in Table A3.

**Table 1:** Summary of the data extraction for the articles included in the review.

Health system component	Adaptation strategy	Targeted extreme weather event/s	Justification for adaptations	Results of adaptations	Authors (year)
Access to essential medicines	Ensuring procurement and supply of medicines during events	Cyclones	Increasing frequency of extreme weather event/s	The examined drug replacement plan could only ensure supply of medicines for events lasting no longer than five days	O'Dwyer et al. [25]
Health information systems	Monitoring extreme weather events, their impacts, and presenting information and warnings to public	Primarily extreme heat, but also bushfires, cyclones, floods, and storms	Previous experience of extreme weather event/s Increasing frequency of extreme weather events	Improved health sector decision making Improved health outcomes (also no benefits for health observed in some cases) Reduced demand on health system Reduced costs	Bell et al. [26] Crompton et al. [27] Nitschke et al. [28] Nitschke et al. [29] Thomson et al. [30] Williams et al. [31]
Health information systems	Assessing health system performance	Primarily bushfires, but also drought, extreme heat, floods, and storms	Previous experience of extreme weather event/s Increasing frequency of extreme weather events	Improved understanding of response requirements Identification of areas within health system that require attention and planning	Reifels et al. [32] Rychetnik et al. [33] Salmon et al. [34]
Health system financing	Securing and mobilising funding	Bushfires	Previous experience of extreme weather event/s	Improved workforce capabilities	Hurst et al. [35]
Health workforce	Workforce training and education	Generally broad and unspecific to a particular extreme weather event	Previous experience of extreme weather event/s Increasing frequency of extreme weather events Perceived lack of preparedness and resilience Need to integrate new practices	Improved workforce understanding of extreme weather event responses Identification of a lack of preparedness for extreme weather events Identification of barriers preventing workforce capabilities	Mohtady Ali et al. [36] Brewer et al. [37] McCourt et al. [38] McCourt et al. [39] Mitchell et al. [40] Scrymgeour et al. [41] Slimings et al. [42] Watson et al. [43] Wild et al. [44]
Leadership and governance	Disaster management plans	Generally broad and unspecific to a particular extreme weather event	Increasing frequency of extreme weather event/s Previous experience of extreme weather event/s	Most current disaster plans are inadequate and insufficiently implemented Need more thorough integration and coordination amongst other components of health system	Chand et al. [45] Chand et al. [46] Loosemore et al. [47] Purcell et al. [48] Purcell et al. [49] Rychetnik et al. [33]
Leadership and governance	Extreme weather-oriented health system planning	Generally broad and unspecific to a particular extreme weather event	Increasing frequency of extreme weather event/s Increasing risks of impacts from extreme weather event/s	Integration and translation of evidence into policy Improved health outcomes	Burton et al. [50] Tonmoy et al. [51]
Service delivery	Maintaining provision of services	Cyclones	Increasing frequency of extreme weather event/s	The examined continued service delivery plan could only ensure continuation of services for events lasting no longer than five days	O'Dwyer et al. [25]
Service delivery	Preparing health infrastructure	Largely broad, but also with a noticeable focus on extreme heat and cyclones	Perceived inadequate infrastructure Increasing frequency of extreme weather event/s Previous experience of extreme weather event/s	Improved resilience of infrastructure Greater capacity to cool surrounds, improve biodiversity, and campus aesthetic Improved health outcomes	de Souza et al. [52] Loosemore et al. [53] Luke et al. [54] Ryan et al. [55] Ryan et al. [56] Ryan et al. [57]

### 3.1 Study characteristics

#### 3.1.1 Study design

Studies were most commonly quantitative in design (*n* = 14), with 12 descriptive cross-sectional studies and one each of randomised and non-randomised controlled trials. Qualitative studies (*n* = 12) used interviews, workshops, focus groups or document analysis to describe and assess their interventions. Finally, some mixed methods studies (*n* = 7) consisted of both designs.

#### 3.1.2 Study year and location

Studies were distributed relatively evenly across the years included in this review, with 12 published between 2014 and 2016, 10 between 2017 and 2020, and 11 between 2021 and 2023. It was most common for articles to not concentrate on an individual location or healthcare organisation, instead examining organisations or healthcare workers across the country (*n* = 8). Of the studies conducted in specific states and territories, the majority were in individual states, including Queensland (*n* = 7), New South Wales (*n* = 6), Victoria (*n* = 3), South Australia (*n* = 3), Northern Territory (*n* = 1), Tasmania (*n* = 1), and Australian Capital Territory (*n* = 1). Two studies drew on multiple states or territories: New South Wales and South Australia [53] and Northern Territory and Queensland [40]. The health systems investigated were also relatively evenly spread across rural and urban locations, with 17 studies reporting on both urban and rural healthcare organisations, seven solely on rural, and nine on those in urban areas.

#### 3.1.3 Quality assessment

The results of the quality assessment indicated that the majority of studies had no or few quality concerns (*n* = 22), while fewer contained omissions that lowered the methodological quality somewhat (*n* = 11). See Table A2 for details of the quality assessment.

#### 3.1.4 Type of extreme weather events

**Fig. 2:**
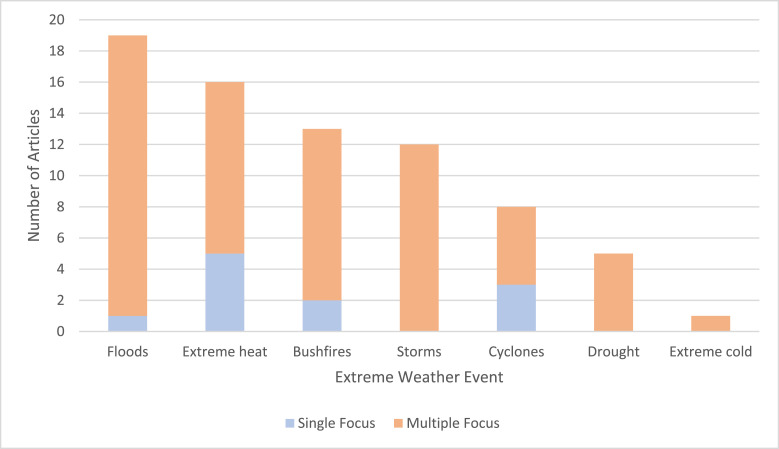
Number of articles identifying a particular extreme weather event as a focus of their adaptation strategy.

Adaptation strategies sought to mitigate seven separately identified EWEs, with most studies identifying adaptations aimed at EWEs generally (*n* = 23); shown in Fig. 2. In these studies, there were specific mentions of floods (*n* = 18), storms (*n* = 12), bushfires (*n* = 11), extreme heat (*n* = 11), droughts (*n* = 5), cyclones (*n* = 5) and extreme cold (*n* = 1). Of the remaining 10 articles, nine identified adaptations that were targeted towards single EWEs, including extreme heat (*n* = 5), cyclones (*n* = 2), and bushfires (*n* = 2), while the adaptation in Crompton et al. was targeted at both floods and cyclones [27].

### 3.2 Adaptation strategies

#### 3.2.1 Across the health system components

**Fig. 3:**
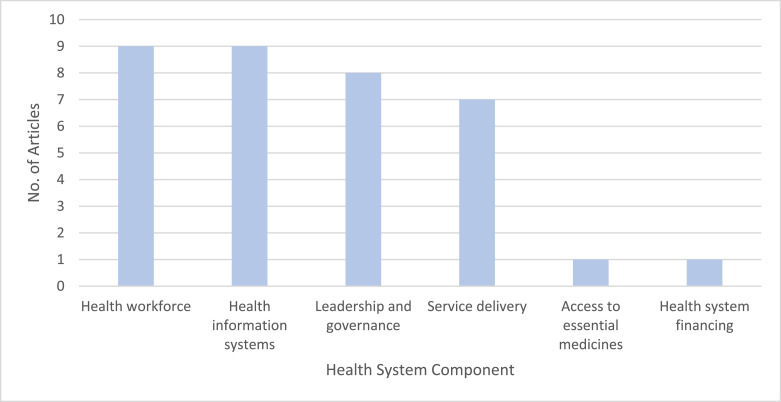
Number of articles identifying adaptation strategies per health system component.

Across the 33 articles included in the review, 31 focused on adaptation strategies within a single component of the WHO's health systems framework [5], while two considered a mixture or combination of adaptation strategies that crossed multiple components of the health system (Fig. 3).

Adaptation strategies relating to the health workforce (*n* = 9) and health information systems (*n* = 9) were the most frequently studied. Health workforce-related strategies included workforce education and training to inform staff about how to act during EWEs and build their sense of preparedness, such as through workshops [43], teaching curricula [44], and drills [36,37]. For health information systems, means of monitoring and evaluating EWEs and their health impacts were most common, including extreme weather warning systems (*n* = 4), tools for assessing the potential capacity of the health workforce to respond to EWEs (*n* = 4), and a health call centre that also acted as a mental health screening tool [27].

It was also common for adaptations to focus on leadership and governance (*n* = 8) and service delivery (*n* = 7). Leadership and governance adaptations included the development and integration of disaster plans into existing organisational structures [[46], [49], [50]]. Adaptations focusing on service delivery mainly intended to improve the resilience of health infrastructure, including measures such as guaranteeing power through backup generators, designing and locating infrastructure to be resilient to extreme weather, preparing the facility's air filtration and cooling, ensuring drinking water can be provided within the health facility reliably, preparing waste, fuel and gas systems, ensuring furnishings are resilient to EWEs [[54], [55], [56], [57]], and providing increased green infrastructure and shaded areas in spaces around healthcare organisations [[52], [53]]. One further study considered service delivery by exploring the provision of an opioid replacement therapy service during EWEs [25].

The least frequently implemented strategies related to access to essential medicines (*n* = 1) and health system financing (*n* = 1). The same article considering opioid replacement therapy also documented steps to ensure access to medical supplies through having sufficient storage and backup options during EWEs [25]. The sole article on health system financing examined how finances are mobilised post-disaster to aid rural health practices [35].

#### 3.2.2 Stage of extreme weather event

Adaptation strategies were implemented at various stages of EWEs. Primarily, adaptations were intended to prepare the health system for future EWEs (*n* = 19), such as running a workshop on disaster preparedness for pharmacists working in hospitals [43] and preparing hospital infrastructure for extreme weather [[28], [54], [55], [56], [57]]. Response-focused adaptations (*n* = 7) included heatwave monitoring and alert programs [[28], [30], [31]], while strategies for recovery (*n* = 4) included grant funding [35] and integrating previous disaster experience into management plans [46,47]. Three articles identified adaptations across multiple stages of EWEs, such as increasing green infrastructure around hospitals and establishing partnerships between hospital staff spanning preparedness and response [[33], [40],^52^].

#### 3.2.3 Scale of implementation

The adaptation strategies were primarily implemented at the local level (*n* = 24), including within hospitals, services within hospitals (such as pharmacies), and networks of hospitals in a local area, often referred to as local health districts. Few strategies were implemented at the state (*n* = 8) and national level (*n* = 2). The national-level adaptations included grant funding from the federal government to promote rural health practice efforts in aiding recovery from bushfires [35] and adding climate change-related health impacts to national general practitioner vocational training curricula [44].

#### 3.2.4 Strategy justification

The adaptations were justified in various ways including, most prominently, having experienced a previous EWE (*n* = 15). Other justifications included the presumed level of risk EWEs posed to the health system in the future (*n* = 8), a perceived need to improve health outcomes resulting from EWEs (*n* = 5), and a perceived lack of preparedness or resilience of the health system (*n* = 4). One article listed a lack of climate-resilient and health-promoting areas around the health organisation that were needed during extreme heat as its justification [52].

### 3.3 Evaluation methods

All included articles evaluated the implemented adaptation. Primarily, studies used one method of evaluation (*n* = 25), while a few adopted multiple methods (*n* = 8). The most popular evaluation methods included surveys (*n* = 11), interviews (*n* = 10), focus groups (*n* = 4) and workshops (*n* = 4) with the health workforce. Utilising hospital health data (*n* = 4), health system observations (*n* = 3), and document analysis (*n* = 2) were also used on multiple occasions. One study used a survey comparing a control and treatment group [28], while another had surface temperature measurements, biodiversity counts, and social media posts [52]. Below, we report on each of these methods in relation to the WHO health system building blocks [5].

#### 3.3.1 Health information systems

Implementations relating to health information systems were evaluated differently depending on whether the focus was on monitoring or evaluation tools. Monitoring tools, such as health alert systems, were evaluated using health data to determine hospital admissions counts [28,30], cost savings [31] and mental health illnesses [27], and via a non-randomised control survey comparing heat stress amongst those who did receive heat health information with a control [29]. However, evaluation tool assessment varied, with a workshop with local government decisionmakers involved in healthcare planning [26], a survey with healthcare workers [32], and document analysis [33,34].

#### 3.3.2 Health workforce

Health workforce adaptations were generally assessed by asking workers how prepared they felt in responding to EWEs or how they rated the adaptation. For instance, quantitative surveys were used to assess the extent to which disaster planning and policies in health systems were associated with workforce preparedness for EWEs (*n* = 3). Similarly, workforce opinions on integrating extreme weather event-related content in health education curricula were gathered using quantitative surveys (*n* = 2). For qualitative evaluation, interviews were used to ask how prepared staff were for EWEs and what inhibited their preparations (*n* = 3). Finally, one article assessed the success of developing partnerships between staff across hospitals to share capacity during EWEs via surveys and focus groups [40].

#### 3.3.3 Leadership and governance

For adaptations relating to leadership and governance, interviews (*n* = 4) and surveys (*n* = 3) of healthcare workers were used to determine how well extreme weather policies and plans were enabling resilience. Healthcare observations (*n* = 2) were used to evaluate vulnerabilities or issues in the implementation and design of disaster management plans.

#### 3.3.4 Service delivery

To evaluate the general service readiness of service delivery for EWEs, including continued service, power and water supply, etc., several articles conducted focus groups with healthcare workers [53,56], along with interviews [55], surveys [57] and observations [54]. Interviews with the health workforce were also used to assess how well opioid replacement therapy services could be maintained during the occurrence of EWEs [25]. De Souza et al. evaluated the effectiveness of green infrastructure around hospitals at reducing temperatures, increasing biodiversity, and improving human comfort through surface temperature measurements, bird surveying, and social media posts [52].

#### 3.3.5 Access to essential medicines and financing

Finally, for adaptations relating to access to essential medicines, interviews were used to determine healthcare workers opinions of how well a ‘dosing in disaster’ plan could allow medical supplies to be continued during EWEs [25]. Similarly, the usefulness of health system financing was evaluated using surveys and interviews to assess workforce capability, professional resilience, mental health and well-being [35].

### 3.4 Outcomes of strategies

#### 3.4.1 Health information systems

Amongst adaptation tools whose primary purpose was to monitor EWEs, heat health alert systems were found to reduce ambulance callouts and emergency presentations [28], heat stress [29] and financial costs [31]. However, Thomson et al. observed no reduction in heat-related morbidity [30] and Nitschke et al. [28] found that mortality was not reduced overall. A state-wide health call centre used to screen mental health illnesses post-EWEs was found to assist in the identification of unmet mental health needs through retrospective analysis of phone call logs [27].

Tools for evaluating health system performance were generally reported by the healthcare workers involved to provide valuable means of understanding and tracking the impacts of EWEs, such as Accimap [34], Composite Capacity Indicators [32], and others [[26], [33]].

#### 3.4.2 Health workforce

Health workforce adaptations typically improved the capacity of the workforce to implement EWE-related practices and adequately respond to EWEs. In hospitals that had disaster plans and policies, the health workforce was found to feel moderately prepared for future EWEs, depending further on whether the workers had experienced previous EWEs and their perceived competence [[37], [38], [39]]. Barriers to increasing preparedness included time limitations, unclear plans, and poor communication across the hospital [41]. A hospital-based disaster preparedness workshop improved participants’ understanding of their disaster management activities and responsibilities post, but the perceived importance of such activities changed little, either positively or negatively [43]. Similarly, introducing planetary health themes to medical programs improved students’ knowledge of how climate change is likely to impact health [42]. Finally, the establishment of a cross-hospital partnership program was found to increase perceived self-preparedness amongst nurses [40].

#### 3.4.3 Leadership and governance

Leadership and governance adaptations were generally found to benefit the health system. Health service managers and practitioners believed that implementing specific EWE plans and policies rendered hospitals more capable of responding to EWEs [[48], [49]]. For instance, planners involved in a collaborative climate adaptation plan across a regional area found that the collaborative plan improved the capabilities of all parties involved [51]. However, shortcomings were also identified in evaluations, including policies in many hospitals inadequately focusing on preparing hospital facilities and amending organisation structures [[46], [50]], and that disaster plans often exist in isolation, focus on man-made disasters, such as terrorism or technological hazards, rather than EWEs, and only involve management rather than those involved directly in health service delivery [[45], [47]].

#### 3.4.4 Service delivery

The adaptations to health system infrastructure to enable general service readiness to EWEs, such as alterations to building design, materials, and shading, tended to improve health system resilience, either according to participants through interviews [55], focus groups [53,56], surveys [57], or through observation [54]. The presence of a ‘dosing in disaster’ plan contributed towards the continuance of opioid replacement therapy services during EWEs by increasing the maintenance capacity of the service, according to healthcare workers [25]. Increasing green infrastructure around the outside of hospitals resulted in cooler temperatures, greater local biodiversity, and improved hospital campus aesthetics [52].

#### 3.4.5 Access to essential medicines and financing

Mixed outcomes were recorded for access to essential medicines and health system financing. Having a ‘dosing in disaster’ plan was noted through interviews to be not enough to ensure continued access to medication in disasters lasting longer than five days [25]. Surveys and interviews with healthcare practitioners revealed that national-level grant funding of rural healthcare air recovery services benefited workforce capability, professional resilience, and mental health and well-being [35].

## 4 Discussion

**Fig. 4:**
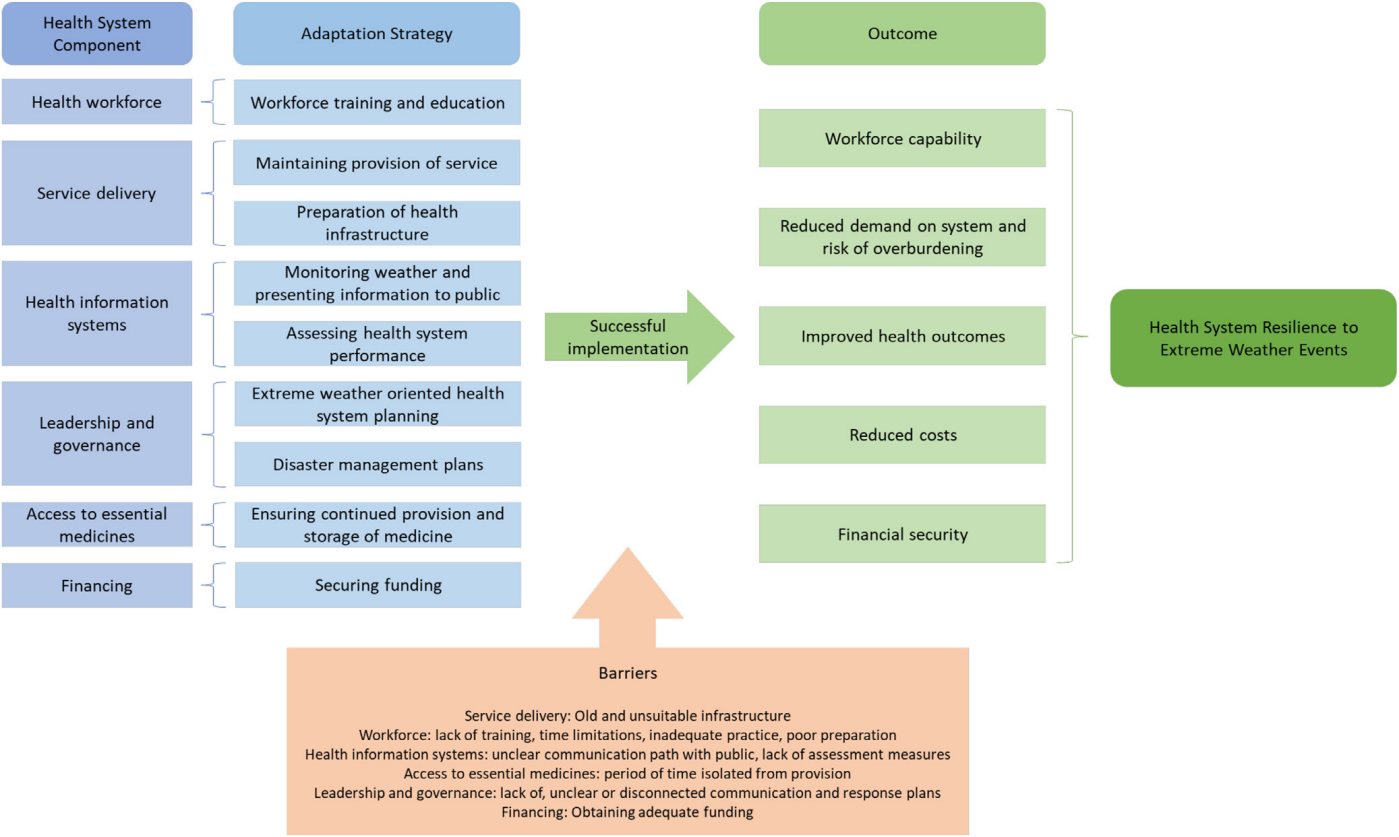
Relationship between the adaptation strategies and outcomes contributing towards health system resilience to extreme weather events.

This review presents the first summary of research investigating the adaptation of the Australian health system to the risks posed by extreme weather. A graphical summary of the documented adaptation strategies and their noted positive outcomes or barriers preventing successful implementation is presented in Fig. 4.

### 4.1 Health system adaptations to extreme weather events

The adaptations identified in this review mostly targeted the health workforce, health information systems, leadership and governance, and service delivery. The adaptations were also primarily preparation-oriented, intending to minimise the impacts of EWEs before they occur. Far less attention was placed on access to essential medicines and health system financing, where adaptations were preparedness and recovery-based, respectively.

This broadly aligns with previous research, such as a review of health system adaptations in the Asia Pacific, which found that the health workforce, service delivery, and leadership and governance were the greatest focal points of adaptations, and that health system financing received the least attention [16]. Contrastingly, however, there was a greater focus on health information systems in Australia, whereas in the Asia-Pacific access to essential medicines received more attention. These discrepancies could reflect the different nature of EWEs in these regions or approaches of the health systems. For instance, health information systems may be more prominent in Australia because of the greater focus on adapting to extreme heat than in the Asia-Pacific. More broadly, it is also possible that older strategies and policies across Australia, such as the *National Strategy for Disaster Resilience* [13], have encouraged focusing more on the health workforce, health information systems, leadership and governance, hence their dominance in Australia's adaptations. The *2023 National Health and Climate Strategy* lists the health workforce and health information systems as priorities, but also more clearly identifies the continuation of health service delivery, which could see more service delivery adaptations implemented in the near future. Given that this review just focused on academic literature, these changes are unlikely to be captured in documented adaptations just yet, meaning that they could already be occurring. Nonetheless, the Australian Government's *National Climate Risk Assessment and National Adaptation Plan* should still pay specific attention to health system finance, access to essential medicines, and ensuring that collaboration across jurisdictions is promoted [13].

The EWEs targeted by adaptations were also somewhat unevenly prioritised. Floods, storms, bushfires and extreme heat were consistently the targets of interventions. Droughts and cyclones were listed less frequently, while extreme cold was only mentioned once. These findings likely reflect Australia's unique climate, whereby floods and bushfires have been particularly prevalent and harmful in recent years [58] and have had documented effects on health systems already [10]. Storms, extreme heat, droughts and cyclones are also common in Australia, although perhaps their impacts have been less prolific or occur in more isolated pockets across the country [59]. Other research on health system resilience to EWEs, such as in the Asia-Pacific region, has also identified floods, storms and cyclones as main priorities and extreme cold as an infrequent concern [16]. In contrast to our present review, however, extreme heat and bushfires were rarely referred to in health system adaptations [16]. Again, this likely reflects the unique risk of these EWEs in Australia, which may be observed in other studies focusing on health systems within similar climates.

### 4.2 Implementation of Australian health system adaptations

Adaptations were primarily implemented at the local level and focused on preparing the health system for predicted future EWEs. Again, it should be emphasised that these trends are reflective of health system adaptations in the literature, and that less visible adaptations currently being developed or recently implemented are not captured here. Nonetheless, given Australia's focus on providing relief and responding to EWEs before 2011 [13], and the lack of a whole-of-government response until the *National Strategy for Disaster Resilience*, it is perhaps unsurprising that the health system adaptations documented in research so far have primarily been responsive in nature and focused on individual health systems, rather than across states or country. It is possible that, as recent policies become more pronounced, the focal points of Australia's health system adaptations will change. It is also possible that adaptations that have not yet been documented in academic literature, because they are so recent or less visible, are already leading to such changes.

### 4.3 Evaluation of health system resilience to future events

While our review highlights the beneficial outcomes of discrete adaptations, including improved workforce capability, better health outcomes, reduced demand and potential overburdening of the health system, lowered costs, and greater financial stability (as displayed in Fig. 4), how those elements come together in an integrated way to build resilience in the health system in alignment with existing frameworks for health system resilience [17] is as of yet not clear and demands further study. Moreover, a number of barriers still remain in the health system that reduce the effectiveness of adaptations which should be the centre of attention to improve resilience going forwards.

### 4.4 Limitations

There are several limitations emerging from this review. First, the wide variety of means of evaluating the identified adaptation strategies makes it difficult to compare the studies included in the review and infeasible to conduct a meta-analysis. Second, as much of this research is documenting health system changes and adaptations that are occurring currently and rapidly, the results reported are likely not up-to-date and do not capture all of the adaptations being implemented across Australia to reduce the risks posed by EWEs. Indeed, adaptations may not necessarily be visible to academics or captured in academic literature and are thus not identified in this present review. Future research should seek to engage more with grey literature and stakeholders in order to form a more complete understanding of health system adaptations in Australia. Further, our finding that response and recovery adaptations are being implemented less than preparative ones, could emerge from difficulties conducting research in the immediate aftermath of EWEs and may not be reflective of current adaptations. Finally, it is difficult to completely assess health system resilience given methodological challenges, as the assessment of methodological quality did find some studies with limitations, and the absence of studies conducted pre- and post-adaptation to EWEs. While a few notable exceptions exist, such as comparing recipients of heatwave health information with a control group [29], more research of this kind is required going forward.

## 5 Conclusion

This review has revealed the range of adaptations documented in academic literature being implemented across Australia's health system to reduce the risks posed by EWEs. The wide variety of methodological approaches and outcomes measured in this body of research, along with some studies with only moderate methodological quality, reflects difficulties in gathering data on health systems and evaluating how well they are adapting to EWEs. We emphasise that more research on and evaluation of adaptation strategies is required, particularly concerning access to essential medicines and health system financing. Future research should also attempt to consolidate approaches and outcomes measured to allow greater comparison across contexts and identify the most effective strategies to build resilience to EWEs.

## CRediT authorship contribution statement

**Rupert Legg:** Writing – review & editing, Writing – original draft, Methodology, Formal analysis, Data curation, Conceptualization. **Jason Prior:** Writing – review & editing, Visualization, Supervision, Methodology, Conceptualization. **Erica McIntyre:** Writing – review & editing, Visualization, Validation, Methodology, Conceptualization. **Edgar Liu:** Writing – review & editing, Visualization, Validation, Supervision, Methodology. **Mikaela Tracy:** Writing – review & editing, Methodology, Formal analysis. **Leona Tan:** Writing – review & editing, Methodology, Formal analysis. **Angela Dawson:** Writing – review & editing, Conceptualization. **John Richmond:** Writing – review & editing, Conceptualization. **Clare Perry:** Writing – review & editing, Methodology, Formal analysis.

## Declaration of competing interest

The authors declare that they have no known competing financial interests or personal relationships that could have appeared to influence the work reported in this paper.

## Appendix Supplementary materials

Image, application 1
