# Supplementary material for: Health system adaptation to extreme weather events in Australia: A scoping review
Source: J Clim Chang Health. 2025 Apr 28;22:100443. doi: 10.1016/j.joclim.2025.100443 (PMC12851259; doi:10.1016/j.joclim.2025.100443)
Supplement: Supplementary file 1 [file mmc1.docx]

**Appendix**

| **Table A1.** | | | |
| --- | --- | --- | --- |
| *Details of the search terms used for the search strategy.* | | | |
| Extreme Weather | Health Systems | Adaptation Strategy | Location |
| Extreme weather | Health system* | Adaptation* | Australia |
| Climate change | Health care | Response* | New South Wales |
| Disaster | Healthcare | Mitigation* | Victoria |
| Catastrophe | Allied health | Preparedness | Tasmania |
| Heat wave* | Health infrastructure | Preparation | Western Australia |
| Heatwave* | Hospital* | Readiness | Queensland |
| Extreme heat | Health service* | Resilience | South Australia |
| Heavy rain* | Health sector |  | Northern Territory |
| Flood* | Health facilit* |  | Australian Capital Territory |
| Flooding | Care service* |  |  |
| Drought* | Health district* |  |  |
| Cyclone* | Health network* |  |  |
| Storm* | Health precinct* |  |  |
| Extreme cold |  |  |  |
| Cold snap* |  |  |  |
| Hail* |  |  |  |
| Bushfire* |  |  |  |
| Wildfire* |  |  |  |
| Forest fire* |  |  |  |

Note: Between columns AND was used, while within OR was used.

| **Table A2.**  *Assessment of Methodological Quality for Included Studies via the MMAT Criteria* | | | | | | | | | | | | | | | | | | | | | | | | | | | | |
| --- | --- | --- | --- | --- | --- | --- | --- | --- | --- | --- | --- | --- | --- | --- | --- | --- | --- | --- | --- | --- | --- | --- | --- | --- | --- | --- | --- | --- |
|  |  | Screening questions | | 1. Qualitative | | | | | 2. Quantitative randomised control trials | | | | | 3. Quantitative non-randomised | | | | | 4. Quantitative descriptive | | | | | 5. Mixed Methods | | | | |
|  | Study | S1 | S2 | 1.1. | 1.2. | 1.3. | 1.4. | 1.5. | 2.1. | 2.2. | 2.3. | 2.4. | 2.5. | 3.1. | 3.2. | 3.3. | 3.4. | 3.5. | 4.1. | 4.2. | 4.3. | 4.4. | 4.5. | 5.1. | 5.2. | 5.3. | 5.4. | 5.5. |
| 1 | Ali 2022 | Y | Y | Y | Y | Y | Y | Y | . | . | . | . | . | . | . | . | . | . | . | . | . | . | . | . | . | . | . | . |
| 2 | Bell 2015 | Y | Y | Y | Y | Y | Y | Y | . | . | . | . | . | . | . | . | . | . | . | . | . | . | . | . | . | . | . | . |
| 3 | Brewer 2019 | Y | Y | . | . | . | . | . | . | . | . | . | . | . | . | . | . | . | Y | Y | Y | CT | Y | . | . | . | . | . |
| 4 | Burton 2014 | Y | Y | Y | Y | Y | Y | Y | . | . | . | . | . | . | . | . | . | . | . | . | . | . | . | . | . | . | . | . |
| 5 | Chand 2015 | Y | Y | Y | Y | Y | Y | Y | . | . | . | . | . | . | . | . | . | . | . | . | . | . | . | . | . | . | . | . |
| 6 | Chand 2016 | Y | Y | Y | Y | Y | Y | Y | . | . | . | . | . | . | . | . | . | . | . | . | . | . | . | . | . | . | . | . |
| 7 | Crompton 2023 | Y | Y | . | . | . | . | . | . | . | . | . | . | . | . | . | . | . | Y | CT | Y | N | Y | . | . | . | . | . |
| 8 | de Souza 2023 | Y | Y | . | . | . | . | . | . | . | . | . | . | . | . | . | . | . | . | . | . | . | . | CT | Y | Y | Y | Y |
| 9 | Hurst 2023 | Y | Y | . | . | . | . | . | . | . | . | . | . | . | . | . | . | . | . | . | . | . | . | Y | N | Y | Y | CT |
| 10 | Loosemore 2014 | Y | Y | Y | Y | Y | Y | Y | . | . | . | . | . | . | . | . | . | . | . | . | . | . | . | . | . | . | . | . |
| 11 | Loosemore 2016 | Y | Y | Y | CT | Y | Y | Y | . | . | . | . | . | . | . | . | . | . | . | . | . | . | . | . | . | . | . | . |
| 12 | Luke 2023 | Y | Y | . | . | . | . | . | . | . | . | . | . | . | . | . | . | . | CT | CT | Y | Y | Y | . | . | . | . | . |
| 13 | McCourt 2021a | Y | Y | . | . | . | . | . | . | . | . | . | . | . | . | . | . | . | Y | CT | Y | N | Y | . | . | . | . | . |
| 14 | McCourt 2021b | Y | Y | Y | Y | Y | Y | Y | . | . | . | . | . | . | . | . | . | . | . | . | . | . | . | . | . | . | . | . |
| 15 | Mitchell 2014 | Y | Y | . | . | . | . | . | . | . | . | . | . | . | . | . | . | . | . | . | . | . | . | Y | Y | Y | Y | Y |
| 16 | Nitschke 2016 | Y | Y | . | . | . | . | . | . | . | . | . | . | Y | Y | Y | CT | Y | . | . | . | . | . | . | . | . | . | . |
| 17 | Nitschke 2017 | Y | Y | . | . | . | . | . | Y | Y | Y | N | Y | . | . | . | . | . | . | . | . | . | . | . | . | . | . | . |
| 18 | O'Dwyer 2020 | Y | Y | Y | Y | Y | Y | Y | . | . | . | . | . | . | . | . | . | . | . | . | . | . | . | . | . | . | . | . |
| 19 | Purcell 2014 | Y | Y | . | . | . | . | . | . | . | . | . | . | . | . | . | . | . | Y | Y | Y | Y | Y | . | . | . | . | . |
| 20 | Purcell 2018 | Y | Y | . | . | . | . | . | . | . | . | . | . | . | . | . | . | . | CT | N | Y | CT | Y | . | . | . | . | . |
| 21 | Reifels 2014 | Y | Y | . | . | . | . | . | . | . | . | . | . | . | . | . | . | . | Y | Y | Y | CT | Y | . | . | . | . | . |
| 22 | Ryan 2016 | Y | Y | Y | Y | Y | Y | Y | . | . | . | . | . | . | . | . | . | . | . | . | . | . | . | . | . | . | . | . |
| 23 | Ryan 2017 | Y | Y | Y | Y | Y | Y | Y | . | . | . | . | . | . | . | . | . | . | . | . | . | . | . | . | . | . | . | . |
| 24 | Ryan 2018 | Y | Y | . | . | . | . | . | . | . | . | . | . | . | . | . | . | . | . | . | . | . | . | N | Y | Y | Y | Y |
| 25 | Rychetnik 2019 | Y | Y | . | . | . | . | . | . | . | . | . | . | . | . | . | . | . | Y | Y | Y | Y | Y | . | . | . | . | . |
| 26 | Salmon 2014 | Y | Y | . | . | . | . | . | . | . | . | . | . | . | . | . | . | . | Y | Y | Y | Y | Y | . | . | . | . | . |
| 27 | Scrymgeour 2020 | Y | Y | Y | Y | Y | Y | Y | . | . | . | . | . | . | . | . | . | . | . | . | . | . | . | . | . | . | . | . |
| 28 | Slimings 2022 | Y | Y | . | . | . | . | . | . | . | . | . | . | CT | Y | N | CT | Y | . | . | . | . | . | . | . | . | . | . |
| 29 | Thomson 2023 | Y | Y | . | . | . | . | . | . | . | . | . | . | . | . | . | . | . | Y | Y | Y | Y | Y | . | . | . | . | . |
| 30 | Tonmoy 2020 | Y | Y | . | . | . | . | . | . | . | . | . | . | . | . | . | . | . | . | . | . | . | . | N | Y | Y | Y | Y |
| 31 | Watson 2020 | Y | Y | . | . | . | . | . | . | . | . | . | . | . | . | . | . | . | Y | N | Y | Y | Y | . | . | . | . | . |
| 32 | Wild 2023 | Y | Y | . | . | . | . | . | . | . | . | . | . | . | . | . | . | . | Y | CT | Y | CT | Y | . | . | . | . | . |
| 33 | Williams 2022 | Y | Y | . | . | . | . | . | . | . | . | . | . | . | . | . | . | . | . | . | . | . | . | CT | Y | Y | Y | Y |
| Note: Ratings were conducted in accordance with the Mixed Methods Appraisal Tool (MMAT; Hong et al., 2018). Abbreviations: Y = Yes, N = No, CT = Can’t tell. S1 = “Are there clear research questions?” S2 = “Do the collected data allow to address the research questions?” 1.1. = “Is the qualitative approach appropriate to answer the research question?” 1.2. = “Are the qualitative data collection methods adequate to address the research question?” 1.3 = “Are the findings adequately derived from the data?” 1.4. “Is the interpretation of results sufficiently substantiated by data?” 1.5. “Is there coherence between qualitative data sources, collection, analysis and interpretation?” 2.1. = “Is randomization appropriately performed?” 2.2. = “Are the groups comparable at baseline?” 2.3. = “Are there complete outcome data?” 2.4 = “Are outcome assessors blinded to the intervention provided?” 2.5 = “Did the participants adhere to the assigned intervention?” 3.1. = “Are the participants representative of the target population?” 3.2. = “Are measurements appropriate regarding both the outcome and intervention (or exposure)?” 3.3. = “Are there complete outcome data?” 3.4. = “Are the confounders accounted for in the design and analysis?” 3.5. = “During the study period, is the intervention administered (or exposure occurred) as intended?” 4.1. = “Is the sampling strategy relevant to address the research question?” 4.2. = “Is the sample representative of the target population?” 4.3. = “Are the measurements appropriate?” 4.4. = “Is the risk of nonresponse bias low?” 4.5. = “Is the statistical analysis appropriate to answer the research question?” 5.1. = “Is there an adequate rationale for using a mixed methods design to address the research question?” 5.2. = “Are the different components of the study effectively integrated to answer the research question?” 5.3. = “Are the outputs of the integration of qualitative and quantitative components adequately interpreted?” 5.4. = “Are divergences and inconsistencies between quantitative and qualitative results adequately addressed?” 5.5. = “Do the different components of the study adhere to the quality criteria of each tradition of the methods involved?” | | | | | | | | | | | | | | | | | | | | | | | | | | | | |

**Table A3.** Data extraction for the articles included in the review.

| **Authors (Year); Study Design** | **State (Location); Setting** | **Extreme Weather Event** | **Adaptation strategy (stage of extreme weather event)** | **Justification for adaptation** | **Scale of adaptat-ion** | **Health system component** | **Adaptation evaluation method** | **Result of adaptation** |
| --- | --- | --- | --- | --- | --- | --- | --- | --- |
| Ali et al. (2022); Qualitative | QLD (Gold Coast and Brisbane); Urban | General  (with specific mentions of bushfires and flooding) | Preparing staff in hospitals for disasters through disaster planning programs (preparedness) | A perceived need to enhance the resilience of hospitals and workforce in the face of climate change impacts and disasters | Local (hospitals) | Health workforce (staff capacity) | Semi-structured interviews with hospital workers (n = 13) asking about the extent to which they are aware of and competent in disaster planning programs | Participants had difficulties adhering to disaster planning and preparedness programs because of time limitations and few opportunities to practice |
| Bell et al. (2015); Qualitative | TAS (statewide); Rural | General (with specific mentions of bushfires, extreme heat, floods, storms) | An information technology-based tool to provide information on health and wellbeing risks support decision-making on local health risks during general climate change impacts (response) | A perceived need for local stakeholders to be able to adequately respond to the impacts of climate change | Local (governments) | Health information systems (monitoring and evaluation) | Stakeholder workshops with local governments and key stakeholders (n = 3) | Participants said the tool could help local governments understand and respond to climate change; participants had concerns about health sector adaptation capacity and community resilience |
| Brewer et al. (2019); Quantitative descriptive | NSW  (statewide); Rural | General (with specific mentions of bushfires, cyclones and floods) | Preparing staff in hospitals for disasters through disaster planning programs (preparedness) | The effects of Cyclone Debbie on regional and rural areas in Queensland and NSW | Local (hospitals) | Health workforce (staff capacity) | Survey of regional and rural emergency nurses (n = 32) | Participants felt moderately prepared for disasters in relation to knowledge, skills and preparation. |
| Burton et al. (2014); Qualitative | Australia (country wide); Rural and Urban | General (with specific mentions of bushfires, extreme heat, floods and storms) | Integrating the potential impacts of climate change into health service planning (preparedness) | Risk of climate change and EWEs going forwards | State (regional agencies responsible for planning health services) | Leadership and governance (policies and practices) | Interviews with health service planners (n = 16) | In its current state, Australia is unprepared for the health impacts of climate change with negative consequences for the health of the Australian population. It needs to start proactively planning health facilities and services to be more resilient |
| Chand et al. (2015); Qualitative | NSW (statewide); Rural | General (with specific mention of floods) | Disaster management planning for improving health infrastructure resilience to EWEs (preparedness) | Previous flood events in New South Wales that have been increasing in frequency and severity | Local (hospitals) | Leadership and governance (policies and practices) | Observations and interviews with stakeholders in the disaster planning and management process (n = 17) | A range of problems with the plans were identified by participants, including: plans exist in isolation from other agencies; focus on man-made disasters; largely compliance driven; top-down and confined to senior and executive management (front-line staff included in planning process) |
| Chand et al. (2016); Qualitative | Australia (country wide); Rural and Urban | General (with specific mentions of heatwaves, floods and storms) | Integrating prior experience of disasters into disaster management planning (recovery) | Having experienced previous EWEs | Local (hospitals) | Leadership and governance (policies and practices) | Interviews with stakeholders in the disaster management process (n = 17) | Several vulnerabilities were identified in relation to hospitals’ sites, built environments and organisational structures |
| Crompton et al. (2023); Quantitative descriptive | QLD (statewide); Rural and Urban | Floods; Cyclones | Statewide mental health screening tool in a health call centre (13HEALTH) during extreme weather event (response) | A perceived need to address the psychosocial health of people affected by floods and cyclones in Queensland | State (regional governments) | Health information systems (monitoring and evaluation) | Quantitative evaluation of records from calls to 13HEALTH  (n = 205,064) | Including the mental health screening program demonstrated opportunistic screening may assist identification of those with unmet mental health needs, although more than 20% declined referral to a mental health specialist |
| de Souza et al. (2023); Mixed methods | NT (Darwin); Urban | Extreme heat | Increasing Green infrastructure in outdoor spaces around hospital (preparedness and response) | The observation that the area surrounding the hospital precinct was hot and did not provide users with restorative and climate-resilient green spaces | Local (hospitals) | Service delivery (general service readiness) | Surface temperature measurements using a thermal imaging camera; Biodiversity measured by conducting bird surveys; Qualitative data gathered by informally collating personal correspondence and social media posts. | Increasing green infrastructure resulted in cooling, greater local biodiversity and improved hospital campus aesthetic |
| Hurst et al. (2023); Mixed methods | NSW  (South Bowenfels and St Leonards); Rural and Urban | Bushfire | Grant funding from the Australian Government and overseen by State Government for rural health practices to aid recovery from disasters (recovery) | Experience of bushfires between 2019 and 2022 | State (regional governments); National (national government) | Health system financing (mobilisation of finances) | Survey and interviews with rural health practitioners  (n = 12) | Recovery funding facilitated improvement in workforce capability, professional resilience, mental health and well-being |
| Loosemore et al. (2014); Qualitative | NSW  (Coffs Harbour) and SA  (Ceduna); Rural | General (with specific mentions of extreme heat, floods, and storms) | A mixture of adaptation strategies identified for hospital infrastructure e.g. materials, shading, structure, design, etc. (preparedness) | Projected EWEs increasing and the risk of health facilities being unable to cope with these EWEs | Local (hospitals) | Service delivery (general service readiness) | Focus group sessions with hospital stakeholders including clinicians, facility managers, staff, representatives (unknown) | While hospital  managers do see hospital infrastructure as important, other elements of the health service are deemed more critical during disasters; Effective adaptations must coordinate across organisational systems. |
| Loosemore et al. (2016); Qualitative | Australia (country wide); Rural and Urban | General (with specific mentions of extreme heat, floods, and storms) | Integrating prior experience of disasters into disaster management planning (recovery) | Having experienced previous EWEs and the risk of future EWEs | Local (hospitals) | Leadership and governance (policies and practices) | Observations and interviews with those involved in disaster drills and disaster planning  (n = 17) | Three major vulnerabilities identified by the results: site and area; built environment; and organisational. |
| Luke et al. (2023); Quantitative descriptive | Australia (country wide); Rural and Urban | General (with specific mentions of extreme heat, floods, and storms) | Hospital facility characteristics that render them resilient to EWEs, such as steel frames, corrugated iron roofs, etc. (preparedness) | Lack of resilience of hospitals in the region to EWEs | Local (hospitals) | Service delivery (general service readiness | Hospital Safety Index Assessments conducted for 6 hospitals through observation of facility infrastructure. | Facilities were disaster resilient, although power and water supply vulnerabilities in health facilities were most common. |
| McCourt et al. (2021a); Qualitative | Australia (country wide); Rural and Urban | General (with specific mentions of bushfires and floods) | Practices pharmacists have implemented to prepare for disasters (preparedness) | Previous impacts of disasters | Local (pharmacies) | Health workforce (staff capacity) | Interviews with pharmacists (n = 13) | 46% of participants reported that they were not prepared for a disaster; Those that were prepared had previous disaster experience |
| McCourt et al. (2021b); Quantitative descriptive | Australia (country wide); Rural and Urban | General (no specific mention of EWEs) | Practices pharmacists have implemented to prepare for disasters (preparedness) | Perceived lack of preparedness of pharmacists to disasters | Local (pharmacies) | Health workforce (staff capacity) | Survey with pharmacists  (n = 123) | How prepared pharmacists felt was dependent on disaster experience, perceived knowledge and skills, colleague preparedness, perceived self-efficacy, previous preparedness behaviours, perceived potential disaster severity, and trust of external information sources |
| Mitchell et al. (2014); Mixed methods | NT (Darwin) and QLD (Brisbane); Urban | General (all) | Partnership between specialised nurses from  geographically disparate hospitals to provide critical support to hospitals in national disasters (preparedness and response) | Increasing frequency of disasters in Australia | Local (hospitals) | Health workforce (staff capacity) | Surveys and focus groups with nurses  (n = 23) | Nurses felt they were well equipped for practice in a partner hospital and the partnership developed professional reciprocity; The partnership was integrated effectively and reflected adequate preparation for disaster response |
| Nitschke et al. (2016); Quantitative non-randomised | SA (Adelaide); Urban | Extreme heat | Heatwave warning program including general heat health advice, heat plan review, and activation of specific and coordination heat plans (response) | Record breaking heatwave in 2009 | State (state governments) | Health information systems (monitoring and evaluation) | Comparing incidence rate  ratios of ambulance callouts,  emergency presentations and mortality data between heatwave and non-heatwave periods | While the total number of ambulance call-outs and emergency presentations were lower in a heatwave after the introduction of the warning program, mortality was not reduced |
| Nitschke et al. (2017); Quantitative RCT | SA  (statewide); Urban | Extreme heat | The provision of heat-health information through Heat Health Messages to combat adverse health effects of heat waves (response) | Frequent heat waves recurring since 2009 | State (state governments) | Health information systems (monitoring and evaluation) | Survey of a control group and recipients of heat-health information (n = 637) | Heat stress was significantly reduced in the intervention group compared to the control |
| O'Dwyer et al. (2020); Qualitative | QLD (Mackay, Rockhampton, Townsville, Yeppoon); Urban and Rural | Cyclones | Implementation and maintenance of opioid replacement therapy (ORT) services during disasters (response) | Increasing frequency of cyclones in Queensland | Local (hospitals) | Service delivery (treatment and patient outcomes);  Access to essential medicines (supply and storage) | Interviews with community pharmacists, hospital pharmacists, and Queensland Opioid Treatment Program employees  (n = 14) | Only Townsville had a detailed ‘Dosing in Disaster’ plan;  Still, the plan was vulnerable to any disaster that would have impacts lasting longer than five days |
| Purcell et al. (2014); Quantitative descriptive | NSW  (rural south-west); Rural | General (with specific mentions of extreme heat, bushfires, storms, and droughts) | Disaster management plans for rural health services to meet surge capacity demands during EWEs (preparedness) | Predicted increasingly negative impacts of climate change on the health of rural Australians | Local (general practitioners and health services) | Leadership and governance (policies and practices) | Survey of general practitioners working in rural health services (n = 68) | 33% to 44% of participants were unsure  that their health service had the capacity to respond to an extreme weather event and may not have been aware of the existence of any plans |
| Purcell et al. (2018); Quantitative descriptive | NSW  (rural south-west); Rural | General (with specific mention of storms, floods, bushfires,  extreme heat and cold) | Disaster management plans for rural health services to meet surge capacity demands during EWEs (preparedness) | Projections of increasing occurrence of EWEs resulting from climate change | Local (health service managers) | Leadership and governance (policies and practices) | Survey of health service managers working in rural health services (n = 43) | Most participants thought their health service could respond to storms  (96%), floods (98%), bushfires (93%), extreme heat (89%) or cold (91%) |
| Reifels et al. (2014); Quantitative descriptive | VIC (statewide); Rural and Urban | General (all) | Development of Composite Capacity Indicators (CCI) to predict disaster mental health workforce capacity (preparedness) | Victorian Black Saturday Bushfires | Local (workers in mental healthcare services) | Health information systems (monitoring and evaluation) | Survey of psychologists, nurses, counsellors, social workers, teachers and community workers  (n = 791) | CCIs provide a methodology for workforce capacity assessment and facilitates disaster preparedness planning, capacity building, and delivery of quality disaster mental health services |
| Ryan et al. (2016); Qualitative | QLD (Townsville); Urban | General (with specific mentions of cyclones, storms and floods) | Preparing public health infrastructure for EWEs through planning and designing for resilience, including: regular updates on health service status, preparing medical supplies, transport and water plans, backup medication, evacuation centres with medical equipment and alternate power supply chains (preparedness) | Recent experience of a number of large scale and devastating cyclones, storms and floods in Queensland | Local (healthcare services); | Service delivery (general service readiness) | Interviews and focus groups with disaster service providers  (n = 40) | The greater the impact of extreme weather on public health service infrastructure, the greater the risks for people with non-communicable diseases |
| Ryan et al. (2017); Qualitative | QLD (Cairns, Darling Downs, Hinterland, Townsville); Rural and Urban | General (with specific mentions of cyclones, floods, and storms) | Preparing public health infrastructure for extreme weather, such as power, sanitation, services, supplies, and  water (preparedness) | Increasing EWEs and their impacts on non-communicable diseases and disruption to public health infrastructure | Local (hospitals and health services); | Service delivery (general service readiness) | Focus groups with environmental health professionals (n = 55) | Less resilient public health infrastructure exacerbates non-communicable diseases during EWEs |
| Ryan et al. (2018); Mixed methods | QLD (statewide); Rural and Urban | General (with specific mentions of cyclones, floods, bushfires, droughts, extreme heat) | Preparing public health infrastructure for extreme weather, such as power, sanitation, services, supplies, and  water (preparedness) | Increasing impacts of EWEs on those suffering from non-communicable diseases | Local (health-care services);  State (government officials and disaster response coordinators) | Service delivery (general service readiness) | Questionnaire (n = 14) and workshop (n = 18) with state government disaster management planners. Survey of people with a non-communicable disease  (n = 552) | Access to water was deemed the most important adaptation strategy according to participants, followed by sanitation and supplies |
| Rychetnik et al. (2019); Quantitative descriptive | NSW (Sydney); Urban | General (with specific mentions of extreme heat, floods, storms, drought) | Implementation of an adaptation assessment model to be used by local health districts to predict and respond to climate change risks (preparedness and response) | Increasing temperatures and heatwaves in Sydney | Local (local health districts) | Health information systems (monitoring and evaluation); Leadership and governance (policies and practices) | Analysis of relevant documents released by state government and local health districts in Sydney | Many local health districts  have existing disaster plans, yet none have incorporated local climate and health adaptation plans |
| Salmon et al. (2014); Quantitative descriptive | VIC (Murrindini); Rural | Bushfires | Implementation of a disaster analysis approach that graphically represents the decisions involved in producing the disaster, called Accimap (recovery) | Murrindindi bushfire | Local (local government) | Health information systems (monitoring and evaluation) | Analysis of documents relating to the bushfire, including predominantly the Victorian Royal Bushfires Commission report on the bushfire response | An Accimap is suited to the analysis of disaster response efforts as it provides a more comprehensive understanding of response performance and better promotes improvements to the overall disaster response system |
| Scrymgeour et al. (2020); Qualitative | Australia (country wide); Rural and Urban | General (with specific mentions of droughts and bushfires) | Preparing nurses in hospitals for disasters through disaster planning programs (preparedness) | The need to harness lessons from previous disasters to minimise future impacts | Local (nurses in hospitals) | Health workforce (workforce capacity) | Interviews with nurses (n = 15) | A range of barriers exist preventing nurses’ preparedness, including problems communication across the hospital, lack of plans to inform crisis decision making, being expected to be resilient to personal impacts |
| Slimings et al. (2022); Mixed methods | ACT (Canberra); Urban | General (with a specific mention of bushfires) | Development and pilot of a planetary health  blended-learning module for a graduate medical program (preparedness) | Lack of integration of planetary health concepts in medical programs | Local (health service) | Health workforce (education) | Quantitative mapping of learning outcomes, measurement of pre- and post-intervention planetary health knowledge, and  a feedback survey with students (n = 46) | Medical students’ knowledge of the impacts of climate change on health increased post intervention |
| Thomson et al. (2023); Quantitative descriptive | VIC (statewide); Rural and Urban | Extreme heat | Heat health alert system (response) | Increasingly warm weather in Victoria and risk of heat waves to health | State (state government) | Health information systems (monitoring and evaluation) | Evaluating heat health alerts correlation with heat-related emergency department presentations and hospital admissions | The heat health alert system was not associated with reductions in heat-related morbidity compared to non-heat health alert periods |
| Tonmoy et al. (2020); Mixed methods | QLD (statewide); Rural and Urban | General (with specific mentions of extreme heat, storms, floods, droughts, cyclones) | Collaborative approach for developing a regional scale climate adaptation plan to be implemented across the state (preparedness) | Impact of previous EWEs | State (state government) | Leadership and governance (policies and practices) | Survey of participants who were also given a discussion paper to read (n = 96), along with three workshops  (n = 71) | Direct engagement with stakeholders enabled translation of evidence into regional policy |
| Watson et al. (2020); Quantitative descriptive | Australia (country wide); Rural and Urban | Cyclones | A workshop on disaster preparation and training for pharmacists working within hospitals (preparedness) | A previous cyclone in Queensland | Local (hospitals) | Health workforce (education) | Workshop with hospital pharmacists (n = 47) | The workshop significantly improved participants understanding of disaster management activities, however, it did little to change their view of its importance |
| Wild et al. (2023); Quantitative descriptive | Australia (country wide); Rural and Urban | General (with specific mentions of bushfires and floods) | Teaching health impacts of climate change integrated with GP vocational training education (preparedness) | Risk posed by climate change to health | National (national general practice organisation) | Health workforce (education) | Questionnaire of general practitioner registrars (n = 879) | Most participants agreed that climate change should be incorporated in training education and GPs should take a leadership role in tackling climate change related issues |
| Williams et al. (2022); Mixed methods | SA (Adelaide); Urban | Extreme heat | Heat health alert system (response) | In response to a heatwave in 2009 that caused mortality and morbidity across south Australia | State (state government) | Health information systems (monitoring and evaluation) | Estimating cost savings by based on reductions in hospital admissions and ambulance callouts | The system averts more spending through reducing admissions than it costs to run |

*Note*. RCT = Randomised Controlled Trial. QLD = Queensland. TAS = Tasmania. NSW = New South Wales. NT = Northern Territory. VIC = Victoria.
